# Templated Sequence Insertion Polymorphisms in the Human Genome

**DOI:** 10.3389/fchem.2016.00043

**Published:** 2016-11-16

**Authors:** Masahiro Onozawa, Peter D. Aplan

**Affiliations:** ^1^Genetics Branch, National Cancer Institute, National Institutes of HealthBethesda, MD, USA; ^2^Department of Hematology, Hokkaido University Graduate School of MedicineSapporo, Japan

**Keywords:** templated sequence insertion polymorphisms (TSIPs), mitochondria, polymorphism, human migration, DNA repair, LINE-1 retrotransposon

## Abstract

Templated Sequence Insertion Polymorphism (TSIP) is a recently described form of polymorphism recognized in the human genome, in which a sequence that is templated from a distant genomic region is inserted into the genome, seemingly at random. TSIPs can be grouped into two classes based on nucleotide sequence features at the insertion junctions; Class 1 TSIPs show features of insertions that are mediated via the LINE-1 ORF2 protein, including (1) target-site duplication (TSD), (2) polyadenylation 10–30 nucleotides downstream of a “cryptic” polyadenylation signal, and (3) preference for insertion at a 5′-TTTT/A-3′ sequence. In contrast, class 2 TSIPs show features consistent with repair of a DNA double-strand break (DSB) via insertion of a DNA “patch” that is derived from a distant genomic region. Survey of a large number of normal human volunteers demonstrates that most individuals have 25–30 TSIPs, and that these TSIPs track with specific geographic regions. Similar to other forms of human polymorphism, we suspect that these TSIPs may be important for the generation of human diversity and genetic diseases.

## Introduction

Maintenance of chromosomal integrity is required for the survival of all organisms, from simple prokaryotes to complex eukaryotes. This maintenance of chromosomal integrity is accomplished by DNA repair enzymes. There are a number of DNA repair systems that operate in eukaryotes, including DNA mismatch repair, DNA single-strand break repair, and DNA double-strand break (DSB) repair. DNA DSB repair can be further subdivided into repair by homologous recombination, “canonical” non-homologous end-joining (NHEJ), and “non-canonical” NHEJ (Chiruvella et al., 2013; Deriano and Roth, 2013).

Transfected plasmid DNA can be captured and used as a patch at the site experimentally induced DNA DSBs; the DNA patches typically show signs of NHEJ, such as micro-deletion, microhomology, and non-templated nucleotide addition (Lin and Waldman, 2001; Varga and Aplan, 2005; Cheng et al., 2010). Moreover, Yu and Gabriel detected mitochondrial DNA fragments at the site of HO endonuclease-induced DNA DSBs (Yu and Gabriel, 1999), demonstrating that a DNA DSB can be repaired by insertion of DNA sequences in yeast.

RNA can provide a template for DNA synthesis during telomere elongation (Autexier and Lue, 2006) or reverse transcription of retrotransposons (Baltimore, 1985). Several lines of evidence suggest that endogenous retrotransposons may also have a role in DNA DSB repair in human cells. When introduced into yeast, the human LINE-1 ORF2 can mediate repair of HO endonuclease-induced DNA DSB via insertion of retrotransposon sequences (Teng et al., 1996), through a cDNA intermediate. Subsequently, synthetic RNA oligonucleotides were shown to be a template for DNA synthesis during repair of HO endonuclease induced DNA DSB in yeast, although the efficiency of repair with RNA oligonucleotides was orders of magnitude lower than DNA oligonucleotides (Storici et al., 2007). Finally, a role for LINE-1 retrotransposons in DNA DSB repair in mammalian cells has been predicted (Morrish et al., 2002, 2007). This prediction was based on clever experiments that showed new integrations of an endonuclease-incompetent LINE-1 retrotransposon could be identified in rodent cell lines; this form of LINE-1 integration was designated endonuclease-independent (ENi) retrotransposition (Morrish et al., 2002). Because these new integration sites lacked the typical features of LINE-1 endonuclease mediated insertions, such as Target-Site Duplications (TSDs) and poly(A) tracts, the authors hypothesized that LINE-1 sequences were used as a “patch” to repair a spontaneous DNA DSB (as opposed to one introduced by the LINE-1 endonuclease).

With the advent of next generation sequencing technologies, millions of germline variants in mammalian genomes within a species have been identified. Most characterized variants, known as polymorphisms, fall into three large categories; single nucleotide polymorphisms (SNPs), small (<50 bp) insertions or deletions, referred to collectively as short indels, and large deletions (>50 bp) (Genomes Project et al., 2012). More recently, a smaller number of polymorphic insertions of retro-elements (such as LINE-1 or Alu) have been identified through the use of sophisticated methods to detect mobile element insertions (Beck et al., 2010; Huang et al., 2010; Iskow et al., 2010). In addition, insertion of processed gene transcripts into the germline have been identified and referred to as polymorphic pseudogenes (Ewing et al., 2013). Recent reports have demonstrated that insertions of retroelements and pseudogenes represent only a fraction of the insertional polymorphisms in the human genome (Onozawa et al., 2014, 2015).

## DNA DSBs can be repaired by insertion of sequences derived from distant regions of the genome in an experimental system

**Figure 1:**
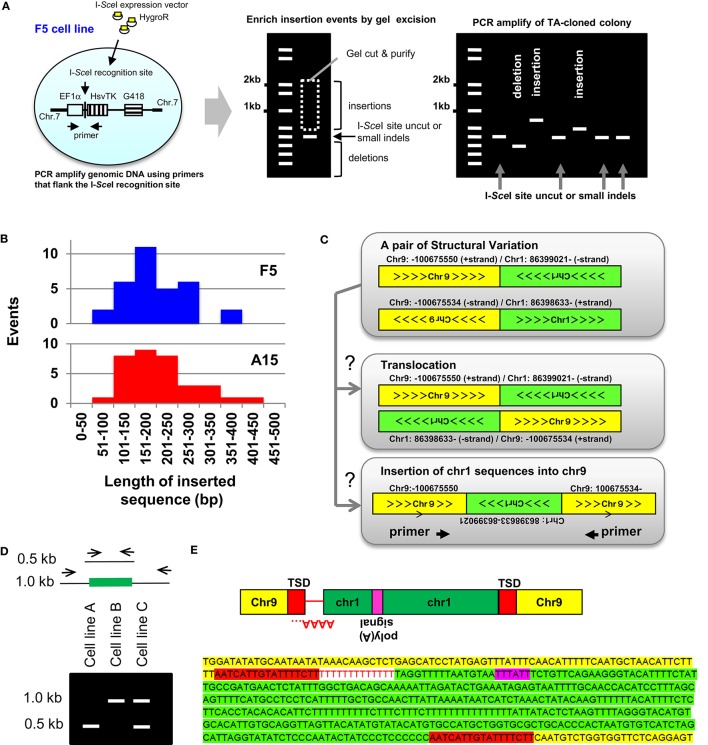
**Insertion mediated repair of DNA DSBs**. (**A**, Left) Outline of the reporter system used to characterize experimental TSIs (Varga and Aplan, 2005; Onozawa et al., 2014). The EF1α promoter (open box), I-*Sce*I recognition sequence, HsvTK cDNA (vertically striped box), and G418R cassette (horizontally striped box) are indicated. (**A**, Middle) Genomic DNA was PCR amplified using primers flanking the I-*Sce*I site. To enrich for PCR fragments containing insertions, the gel portion containing fragments of 0.5–2.0 kb was purified, ligated into plasmids, and inserts from individual colonies PCR amplified. (**A**, Right) Schematic of result showing colonies containing insertions, small indels, and deletions (Onozawa et al., 2014). **(B)** Size of insertions events recovered from F5 and A15 cell lines varied from 73 to 414 bp (median, 191 bp) (Onozawa et al., 2014). **(C)** Identification of insertions from whole-genome sequence data. SV data shows chromosome-9 sequences fused to chromosome 1 and reciprocal chromosome 1 sequences fused to chromosome 9. Sequence fragments are consistent with either a balanced translocation or an insertion of a chromosome 1 sequence into chromosome 9 (Onozawa et al., 2014). **(D)** Analysis of candidate TSI (schematic). PCR primers anneal to TSI acceptor locus (for example, chr 9 from Panel **C**). Amplification of a TSI leads to a larger (1.0 kb) PCR fragment, as shown. Presence of identical insertion-containing 1.0 kb PCR fragments in independent cell lines (cell line **B** and **C**) suggests an insertional polymorphism, which can be confirmed by nucleotide sequence analysis (Onozawa et al., 2014). **(E)** Nucleotide sequence of the insertion shown in Panels **C,D**. Chromosome 9 sequences, target-site duplication (TSD), poly(A) tail (negative strand), polyadenylation signal, and chromosome 1 insertion are indicated. Figure modified from Onozawa et al. (2014).

Templated Sequence Insertions (TSI) were first characterized using a cell culture based approach to study DNA DSB (Varga and Aplan, 2005). These studies modified an experimental system that had been popularized by Jasin and colleagues (Jasin and Haber, 2016), and employed a vector (designated EF1αTK), that contained the EF1α promoter driving expression of the herpes simplex virus thymidine kinase (HsvTK); the recognition site for the rare-cutting meganuclease I-*Sce*I was inserted between the EF1α promoter and HsvTK cDNA (Varga and Aplan, 2005). Expression of the HsvTK enzyme in mammalian cells confers sensitivity to the nucleoside analog ganciclovir; thus, millions of cells can quickly be screened for loss of HsvTK expression by treatment with ganciclovir. The EF1αTK vector was electroporated into the human leukemia cell line U937, or ovarian cancer cell line OVCAR8. Sub-clones that had integrated a single copy of the EF1αTK vector, designated “F5” for U937 (Varga and Aplan, 2005) or “A15” for OVCAR8 (Cheng et al., 2010), were isolated (Figure 1A). In an attempt to induce gross chromosomal rearrangements, an I-*Sce*I expression vector was introduced into the cell lines, followed by ganciclovir (GCV) selection of clones that had lost expression of HsvTK due to mis-repair of a DNA DSB.

Although most of the GCV-resistant clones had short deletions encompassing the HsvTK start codon, rare clones that had undergone an insertion at the DNA DSB site were identified (Varga and Aplan, 2005; Cheng et al., 2010; Onozawa et al., 2014). In these clones, the inserted sequence was not derived from nearby genomic sequences, but instead mapped to a distant region of the genome. After modifying the procedure to enrich for insertions (Figure 1A), 32 insertions of sequences derived from distant regions of the genome were identified in F5 subclones and 34 in A15 subclones (Figure 1B). The origin of these insertions mapped to 18 of 24 human chromosomes, without an obvious preference for any chromosome or chromosomal region (Onozawa et al., 2014). These insertions were designated “TSI,” or TSI, in contrast to the short, non-TSI seen at the site of NHEJ-mediated repair of a DNA DSB (Onozawa et al., 2014). The TSI junctions often showed features of NHEJ, such as microhomology and non-templated nucleotide addition. Generation of TSIs seemed to be generalizable, as they were not restricted to I-*Sce*I induced cleavage but also found at TALEN-mediated cleavage sites (Onozawa et al., 2014).

## TSIs are derived from RNA

The TSIs inserted at the site of NHEJ mediated DNA DSB were not excised from the genome, since the donor sites were intact, and the donor (inserted) sequence had an additional copy compared to genomic regions flanking the donor site (Onozawa et al., 2014). The TSI sequences were enriched for transcribed sequences, suggesting that the TSI may have originated via reverse transcription of RNA. In addition, treatment of cells that expressed the I-*Sce*I enzyme with reverse-transcriptase inhibitors suppressed the frequency of TSIs at DNA DSB site by more than three-fold. Moreover, co-transfection of murine RNA with an I-*Sce*I expression vector into the human F5 cell line showed that reverse transcribed murine sequences were used as insertions at the I-*Sce*I cleavage site. Finally, three insertions displayed mammalian telomere repeat (TTAGGG)n sequences, suggesting that telomerase RNA can also be used as a TSI template. Taken together, although other mechanisms, such as template switching by DNA polymerases or break-induced repair remain possible (Malkova and Haber, 2012; Morrish et al., 2013), the above observations support the hypothesis that reverse transcribed RNA can be used as a template to patch a DNA DSB (Onozawa et al., 2014). Potential sources of this reverse transcriptase activity include LINE-1 ORF2, HERVs (Hohn et al., 2013), as well as the TERT subunit of telomerase, which has recently been suggested to possess hTR-independent RT activity (Sharma et al., 2012).

## TSIs are not an artefact of experimental, induced DNA DSB, but can be identified in unmanipulated human cells

TSIs cannot be detected from routine analysis of whole genome sequence (WGS) reads, which are typically <150 bp. However, TSIs can be identified from WGS using the principles outlined below. First, the junction of two non-homologous chromosomes is designated as a “structural variant” (SV) on short read WGS; pairs of SVs that map very closely can be ascertained by inspection of SV files (Figure 1C). For a pair of SVs to represent a Templated Sequence Insertion Polymorphism (TSIP), both fusion junctions must be located within 50 kb of one another. Second, the strand polarity must align such that an insertion is feasible. Third, each end of the SV needs to be localized to a single, unique genomic loci; SV that show multiple or imperfect alignments (<95% sequence identity) are excluded. Fourth, all highly repetitive alpha satellite sequences are discarded.

The authors screened SVs obtained from two multiple myeloma cell lines (KP6, MC1286PE1) using these criteria and identified 23 unique, verified TSIs (Onozawa et al., 2014). A typical example is shown in Figures 1C–E. Briefly, although this pair of SVs is consistent with a balanced translocation, it is also consistent with an insertion of chromosome 1 sequences into chromosome 9 (Figure 1C). Primers were generated that could amplify the putative insertion, including flanking sequences and both junctions, on a single PCR fragment (Figures 1D,E). Nucleotide sequence of the PCR product verified that the SVs were indeed produced by insertion of chromosome 1 derived sequences into chromosome 9, as opposed to a reciprocal translocation between chromosomes 1 and 9 (Figure 1E). Interestingly 8 out of 23 insertions were identical or nearly identical in the two cell lines (Onozawa et al., 2014) (a schematic example of this phenomenon is shown in Figure 1D), suggesting that these insertions represent polymorphisms in the human genome as opposed to SV acquired by the tumor cells. Consistent with the TSI definition above, these polymorphic insertions were designated templated-sequence insertion polymorphisms (TSIPs).

## Most TSIs identified in normal human subjects are polymorphic

A publicly available database of WGS from 52 normal individuals of defined ethnic/geographic groups (“SV baseline genome set,” filename B37baselinejunctions.tsv, available at http://www.completegenomics.com/sequence-data/download-data/) contained a total of 39,595 SVs from the 52 individuals (Onozawa et al., 2015). Using the criteria set forth in section IV above, 171 candidate TSIPs were identified (Onozawa et al., 2015). Each individual had an average of 25–30 TSIPs, and TSIPs could be classified as “common” (26%; present in at least 20% of individuals), or “rare” (74%; present in <20% of individuals). Interestingly, three TSIPs had a frequency of almost 100%, suggesting that the reference human genome (GRCh37/hg19) is based on an uncommon variant that lacks these three TSIPs. When divided into four regional “super groups” of specified geographic origin (African, Asian, European, North American), common TSIPs were present in individuals from all regions, whereas rare TSIPs tended to be restricted to individuals from a single region (Onozawa et al., 2015). There were more TSIPs per individual of African origin than other geographic regions. All of these findings are consistent with diversity identified in previous studies of mitochondrial and Y-chromosome sequences, and are consistent with patterns of human migration and the hypothesis that *Homo sapiens* originated in Africa (Cann et al., 1987; Hammer, 1995; Underhill et al., 2000).

The investigators obtained genomic DNA from eight of the normal individuals, who had a total of 89 candidate TSIs and successfully validated 69/89 (77.5%) candidate TSIPs (Onozawa et al., 2015). Since these insertions can be polymorphic, they must be heritable, leading to the conclusion that the insertion event must have taken place in either a germ cell (sperm or egg), or early-stage embryo.

## Sequences used as templates for TSIPs

Nucleotide sequence analysis of the insertion sequences revealed that partial LINE-1 elements, cDNAs (with several spliced exons), non-annotated intergenic or intronic sequences, and mitochondrial sequences were used as templates for TSIPs identified in normal individuals (Onozawa et al., 2015). Of note, although mitochondrial fragment insertions were commonly identified as TSIP donor sequences, no TSIs derived from mitochondrion were identified in experimentally induced TSIs using the F5 and A15 cell lines described above (Varga and Aplan, 2005; Cheng et al., 2010; Onozawa et al., 2014). Although speculative, it is possible that mitochondrial sequence insertions may be reproduction-specific events that take place in germ cells or early stage embryos, but do not occur, or occur only rarely, in somatic cells. Interestingly, sperm mitochondria are known to be ubiquitinated and destroyed shortly after fertilization (Sutovsky et al., 2000), leading to the hypothesis that fragmented paternal mitochondrial DNA [or reverse transcribed RNA that was encoded by mitochondrial DNA (Sharma et al., 2012)] can be used to patch a DNA DSB in a fertilized embryo, leading to a TSIP which contained mitochondrial sequence in all cells of the individual, including germ cells (Woischnik and Moraes, 2002; Onozawa et al., 2015; Zhou et al., 2016). Although no TSIPs contained telomere sequences (Onozawa et al., 2014, 2015), interstitial telomeric sequences (ITSs) have been identified in several species (Ruiz-Herrera et al., 2002), and were identified as insertions in the I-*Sce*I experimental system (Onozawa et al., 2014), leading to the speculation that the ITSs may have resulted from telomere patches used to repair a DNA DSB (Nergadze et al., 2004, 2007; Onozawa et al., 2014).

## Class 1 and class 2 TSIPs

**Figure 2:**
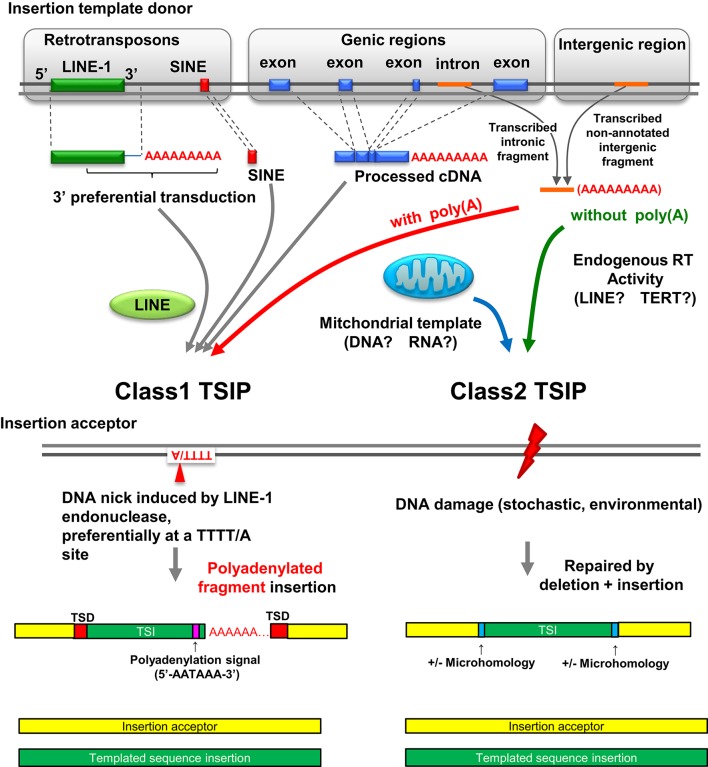
**Landscape of insertion polymorphisms in the human genome**. LINE-1 mediated integration of LINE-1/SINE sequences, LINE-1 sequences (which may include additional 3′ transduced sequences), and processed cDNA insertions are known to create insertion polymorphisms (Beck et al., 2010; Huang et al., 2010; Iskow et al., 2010; Ewing et al., 2013). Polyadenylated intronic or intergenic fragments can also be acted upon in *trans* by LINE-1 ORF2 and integrate at the site of a nick created by LINE-1 ORF2, resulting in a class 1 TSIP. Class 2 TSIPs can be generated by reverse transcription of RNA transcripts into a cDNA patch that is used to repair a DNA DSB via a NHEJ mechanism. Alternatively, RNA could be inserted in the DNA DSB and used directly as a patch template, as reported for yeast (Storici et al., 2007). Finally a DNA DSB can be repaired by fragments of mitochondrial DNA or cDNA; mitochondrial insertions seem to be unique to germ cells or embryos. Figure modified from Onozawa et al. (2015).

TSIPs can be placed into two classes (class 1 and class 2) based on nucleotide sequences at the insertion site (Figure 2). Class 1 TSIPs show a duplication of recipient sequences at both insertion junctions of at least 5 bp; this feature is reminiscent of a TSD that is characteristic of insertions caused by cleavage and insertion of LINE-1 sequences. In addition, class 1 TSIs typically inserted at a preferred LINE-1 integration site (consensus sequence 5′-TTTT/A-3′), and contained a non-templated addition of 10–40 “A” residues, as well as a polyadenylation signal (5′-AATAAA-3′) located 10–20 nucleotides upstream of the poly(A) track. These features strongly support an insertion mediated by LINE-1 ORF2 protein, which contains both endonuclease and reverse transcriptase activity, acting upon non-LINE-1 RNA, and inserting the sequence into a distant region of the genome (Luan et al., 1993; Piskareva et al., 2013). Moreover, since these events must have occurred in germ cells or embryos to be transmitted, it is interesting to note that LINE-1 elements are de-repressed and active in embryos (Castro-Diaz et al., 2014). We can detect no obvious physiologic function for the Class 1 TSIPs, and suspect that these are caused by a careless LINE-1 ORF2 protein causing mischief throughout the genome. Class 2 TSIPs had none of these features (i.e., no TSDs, cryptic poly(A) signal, or poly(A) tract) but instead displayed NHEJ features such as microdeletion, microhomology, and non-templated nucleotide addition at the insertion junction, similar to what one would predict if an ENi retrotransposition event (Morrish et al., 2002) used non-LINE-1 RNA as a template. Consistent with this prediction, it is well established that LINE-1 ORF2 can act in *trans* (Wei et al., 2001). All experimental DNA DSB repair events were class 2 events, and we speculate that class 2 TSIPs are caused by DNA DSB repair events that occurred in a germ cell or early stage embryo of an ancestral individual.

## Potential to cause genetic disease

There is potential for this mechanism of DNA DSB repair to cause genetic disease. Several TSIPs disrupted the coding region of a gene (Onozawa et al., 2015). Furthermore, a recent report described a constitutional 72-bp insertion of mitochondrial sequence into the coding region of *GLI3*, leading to Pallister-Hall syndrome (Turner et al., 2003). Of note, the conception of this patient was temporally and geographically associated with high-level radioactive contamination following the Chernobyl accident (Turner et al., 2003). Although speculative, it is conceivable that a DNA DSB in the germ cell, caused by ionizing radiation, was repaired by a TSI derived from mitochondrial DNA in this individual.

## Conclusion

TSIPs encompass several forms of insertion polymorphisms in human genomes, and are mediated via a combination of mechanisms. Class 1 TSIPs are retrotransposon-mediated events that insert polyadenylated, reverse-transcribed cDNA into seemingly random regions of the genome, whereas class 2 TSIPs are consistent with DNA DSB repair events, in which a short fragment of reverse-transcribed RNA is used as a patch to repair a DNA DSB. These TSIPs provide unique polymorphic markers, similar to SNPs and variable tandem repeats, and can be used to track population migration and evolution. Similar to retrotransposon insertions, we suspect that TSIPs, which can disrupt coding regions of the genome, may play a role in both the etiology of genetic diseases as well as mammalian evolution.

## Author contributions

PA conceived and edited mini-review. MO conceived mini-review, generated figures, and wrote the first draft.

### Conflict of interest statement

The authors declare that the research was conducted in the absence of any commercial or financial relationships that could be construed as a potential conflict of interest.
